# Control of β-glucan exposure by the endo-1,3-glucanase Eng1 in *Candida albicans* modulates virulence

**DOI:** 10.1371/journal.ppat.1010192

**Published:** 2022-01-07

**Authors:** Mengli Yang, Norma V. Solis, Michaela Marshall, Rachel Garleb, Tingting Zhou, Daidong Wang, Marc Swidergall, Eric Pearlman, Scott G. Filler, Haoping Liu

**Affiliations:** 1 Department of Biological Chemistry, University of California, Irvine, California, United States of America; 2 School of Pharmacy & Pharmaceutical Sciences, University of California, Irvine, California, United States of America; 3 Division of Infectious Diseases, Lundquist Institute for Biomedical Innovation at Harbor-UCLA Medical Center, Torrance, California, United States of America; 4 Department of Physiology and Biophysics, University of California, Irvine, California, United States of America; 5 Amgen Inc. Thousand Oaks, California, United States of America; 6 David Geffen School of Medicine at UCLA, Los Angeles, California, United States of America; 7 Institute of Immunology, University of California, Irvine, California, United States of America; University of Exeter, UNITED KINGDOM

## Abstract

*Candida albicans* is a major opportunistic pathogen of humans. It can grow as morphologically distinct yeast, pseudohyphae and hyphae, and the ability to switch reversibly among different forms is critical for its virulence. The relationship between morphogenesis and innate immune recognition is not quite clear. Dectin-1 is a major C-type lectin receptor that recognizes β-glucan in the fungal cell wall. *C*. *albicans* β-glucan is usually masked by the outer mannan layer of the cell wall. Whether and how β-glucan masking is differentially regulated during hyphal morphogenesis is not fully understood. Here we show that the endo-1,3-glucanase Eng1 is differentially expressed in yeast, and together with Yeast Wall Protein 1 (Ywp1), regulates β-glucan exposure and Dectin-1-dependent immune activation of macrophage by yeast cells. *ENG1* deletion results in enhanced Dectin-1 binding at the septa of yeast cells; while *eng1 ywp1* yeast cells show strong overall Dectin-1 binding similar to hyphae of wild-type and *eng1* mutants. Correlatively, hyphae of wild-type and *eng1* induced similar levels of cytokines in macrophage. *ENG1* expression and Eng1-mediated β-glucan trimming are also regulated by antifungal drugs, lactate and N-acetylglucosamine. Deletion of *ENG1* modulates virulence in the mouse model of hematogenously disseminated candidiasis in a Dectin-1-dependent manner. The *eng1* mutant exhibited attenuated lethality in male mice, but enhanced lethality in female mice, which was associated with a stronger renal immune response and lower fungal burden. Thus, Eng1-regulated β-glucan exposure in yeast cells modulates the balance between immune protection and immunopathogenesis during disseminated candidiasis.

## Author summary

*Candida albicans* is a major cause of invasive candidiasis, which has a high mortality rate. It is a constituent of the human microbiome and found in the gastrointestinal and genitourinary tracts of most healthy individuals. *C*. *albicans* is able to switch reversibly between yeast and hyphae in response to environmental cues, and the ability to switch is essential for virulence. The innate immune response is critical for host defense against fungal infections. However, the relationships between morphogenesis, host immune recognition and immune protection are not fully understood for *C*. *albicans*. This study shows that β-glucan is masked by two highly expressed yeast proteins, the endo-1,3-β-glucanase Eng1 and Yeast Wall Protein 1 Ywp1. β-glucan is exposed during yeast-to-hypha transition. Eng1 level is also dynamically regulated in response to different carbon sources and antifungal drugs. The *eng1* mutant modulates virulence in the mouse model of hematogenously disseminated candidiasis in a sex and Dectin-1-dependent manner. Our data reveal that the level of β-glucan exposure in *C*. *albicans* is highly controlled to modulate the balance between immune protection and immunopathogenesis during disseminated candidiasis.

## Introduction

*Candida albicans* is a major opportunistic fungal pathogen of humans. Systemic candidiasis is the fourth leading cause of nosocomial bloodstream infections in the United States [1,2]. The mortality of patients with invasive candidiasis exceeds 40% even with antifungal treatment. The innate immune response is critical for the host defense against invasive candidiasis, but a dysregulated immune response can also be detrimental by causing tissue damage during sepsis [3–5]. It is critically important to understand how *C*. *albicans* regulates recognition by host immune cells to modulate the host immune response.

The host immune response to candidal infection is initiated when pattern-recognition receptors (PRR) on innate immune cells recognize *Candida* cell wall carbohydrates, which serve as pathogen-associated molecular patterns (PAMPs). A major PRR for fungi such as *C*. *albicans* is Dectin-1, a C-type lectin-receptor that can recognize β-1,3-glucan on the fungal cell wall and is required for the host defense against hematogenously disseminated candidiasis [6,7]. The *C*. *albicans* cell wall consists of an outer layer of mannosylated proteins and an inner layer of mostly β-1,3-glucan and underlying chitin [8]. β-1,3-glucan is partially masked from Dectin-1 detection by the outer mannan layer [8,9]. β-glucan exposure on the cell wall is regulated by many factors, including growth conditions, such as lactate, hypoxia, iron limitation and acidic pH [10–14]. β-glucan exposure is also elevated in mutants with defects in glycosylation and mannan synthesis [15], with antifungal treatment [16,17], and is dependent on *C*. *albicans* strain backgrounds [18]. However, the molecular mechanisms by which *C*. *albicans* controls β-glucan exposure in response to different growth conditions are still obscure. Several signaling pathways have been shown to modulate β-glucan exposure, including the Cek1 MAP kinase pathway [12,19–21]. Upregulation of Cek1, which leads to β-glucan unmasking and increased production of proinflammatory cytokines *in vitro*, is correlated with decreased organ fungal burden and virulence in a mouse model of systemic infection. Recently, the exoglucanase Xog1 is shown to function downstream of the lactate-induced β-glucan “masking” pathway through an epitope shaving mechanism [21]. However, downstream targets of the signaling pathways that control β-glucan exposure in many conditions have yet to be identified.

*C*. *albicans* can grow as yeast, pseudohyphae and hyphae *in vitro* and *in vivo* depending on environmental conditions. The ability to transition reversibly between yeast and hyphae is critical for its virulence [22]. Yeast cells are important for dissemination, while hyphae facilitate host invasion and damage [8]. Several studies have shown differential immune recognition and cytokine responses of myeloid cells to *C*. *albicans* yeast and hyphae. Yeast-locked mutants are defective in inducing IL-1β production [23]. Hyphae activate the NLRP3 inflammasome significantly more than yeast, resulting in increased production of bioactive IL-1β [24–26]. Hyphae secrete candidalysin, a peptide toxin that induces inflammasome activation during invasion [27,28]. The increased inflammasome activation/IL-1β production in response to hyphae is partially dependent on Dectin-1, suggesting an increased recognition of β-glucans in hyphae [29]. On the other hand, Mukaremera *et al*. reported that hyphae stimulated proportionally lower levels of cytokines per unit of cell surface area than yeast [30]. In addition, some mutants that are not defective in hyphal morphogenesis are still impaired in inflammasome activation [31,32]. Determining levels of immune response to yeast and hyphal cells *in vitro* is complicated by non-synchronous yeast-to-hyphae transition and interactions with immune cells, as well as aggregation of hyphae. Whether hyphae have greater exposures of β-glucan and Dectin-1 recognition is also controversial [33–35]. Although Gantner *et al*. reported that β-glucan exposure was detectable only on yeast cells at the bud scar but not on hyphae [33], recent investigations observed Dectin-1 binding to hyphae [34,35]. Furthermore, the yeast wall protein Ywp1 is found to contribute to β-glucan masking [34]. Despite some correlations between virulence and innate immune activation in clinical isolates of *C*. *albicans* [36], the molecular link(s) between β-glucan masking, virulence and dimorphism is missing.

β-glucan masking by means of epitope shaving was first shown in *Histoplasma capsulatum*, which secretes an endo-1,3-β-glucanase (Eng1) to trim exposed β-glucan from the cell surface to prevent Dectin-1 recognition [37]. Whether Eng1 is used by *Candida* species to modulate β-glucan exposure is not clear. Here, we show that β-glucan is masked in yeast and exposed during hyphal development. The endo-1,3-glucanase Eng1 is differentially expressed in yeast, and together with the Yeast Wall Protein Ywp1, controls β-glucan exposure and Dectin-1 dependent immune activation. In addition to regulation during the yeast-to-hypha transition, *ENG1* expression is highly regulated and is responsible for β-glucan protection and immune recognition in response to different carbon sources and drug treatments. The *eng1* mutant modulates virulence in the model of hematogenously disseminated candidiasis in a Dectin-1 dependent manner. This study provides a molecular link between yeast-hyphal transition, β-glucan protection and virulence.

## Results

### β-glucan is masked in yeast and exposed during the yeast-to-hyphae transition

Here, we directly compared Dectin-1 binding between yeast and hyphae, using Dectin-1-Fc and a fluorescein-conjugated secondary antibody. Hyphae were induced and grown in YPD at 37°C for 1 and 5 hours, or in RPMI for 5 and 24 hours. Yeast cells were grown at 30°C for the same length of time and in the same medium for comparison.

**Fig 1:**
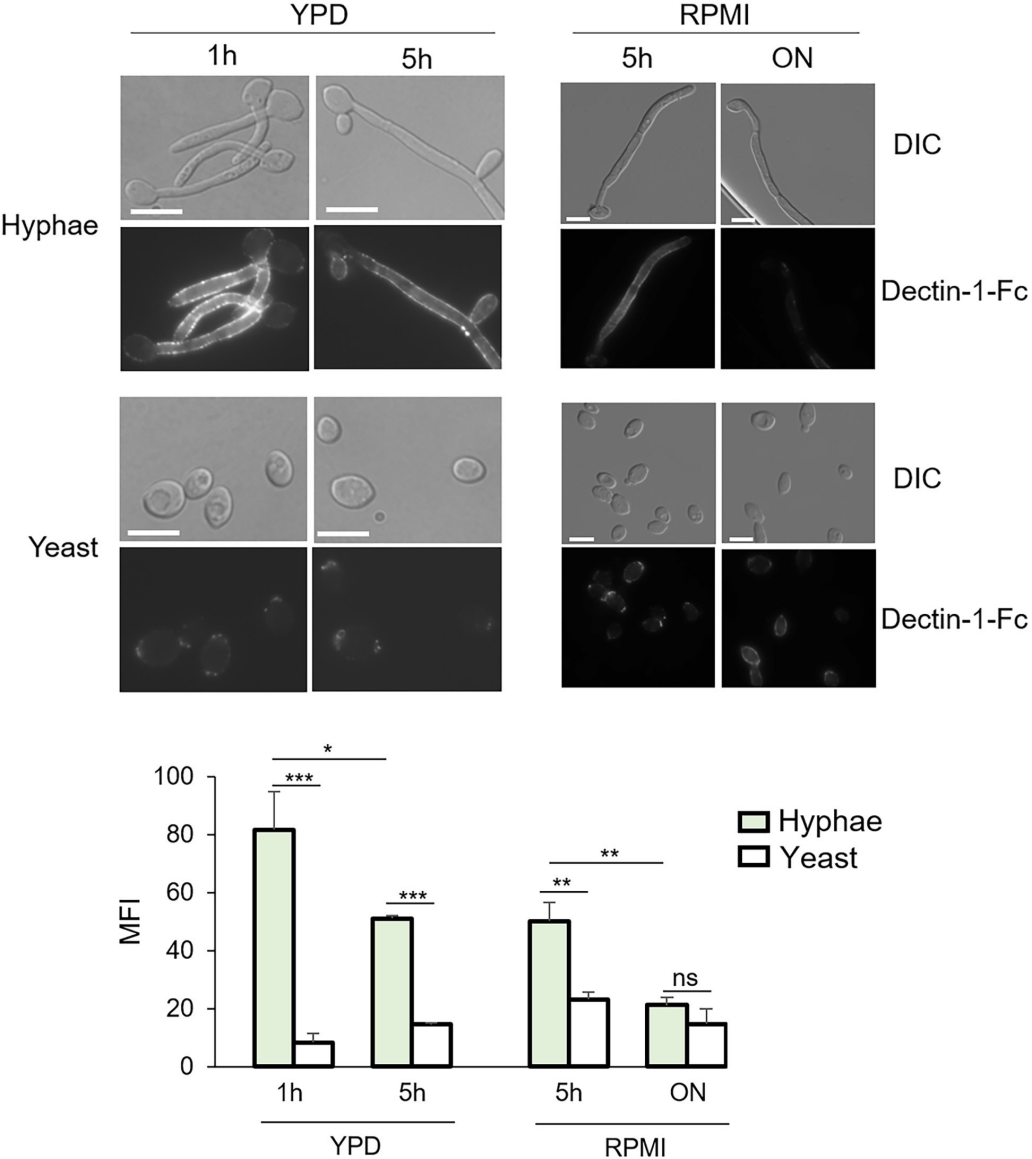
β-glucan is masked in yeast and exposed in germ-tubes. Representative images of *C*. *albicans* cells stained with Dectin-1-Fc and secondary antibody conjugated to FITC. Hyphae were induced at 37°C for indicated hours and in indicated media. Yeast cells were cultured at 30°C in same medium and for same length of time as hyphae. A fixed time of exposure was used in image acquisitions for all pictures. Experiments were repeated three times, The scale bar represents 10μM. Mean fluorescence intensities per area were quantitated by ImageJ. *p* Values were calculated using one-way ANOVA with Tukey post hoc analysis (***p<0.001, **p<0.01, *p<0.05).

Dectin-1 binding was observed on the cell wall of germ tubes (1 h in YPD) and hyphae at 5 h in YPD and RPMI, but not on hyphae from overnight RPMI or the basal yeast cells (Fig 1). Yeast cells grown in the same medium showed Dectin-1 binding only at the bud scar as reported previously [33] (Fig 1). The signal was specific to Dectin-1 binding of β-glucan as adding laminarin (soluble β-glucan) inhibited Dectin-1 binding to hyphae (S1A Fig). The absence of detectable Dectin-1 binding on hyphae from 24 h in RPMI is consistent with the observation made by Gartner *et al*. [33]. Our results show that β-glucan is masked in yeast and exposed in germ-tubes during hyphal initiation. β-glucan exposure on hyphae is also affected by the hyphal growth stages, which explains the variable results from different publications [33–35]. How β-glucan masking is differentially regulated in hyphae and yeast is not clear.

### The endo-1,3-glucanase Eng1 is differentially expressed in yeast to control β-glucan exposure in *C*. *albicans*

**Fig 2:**
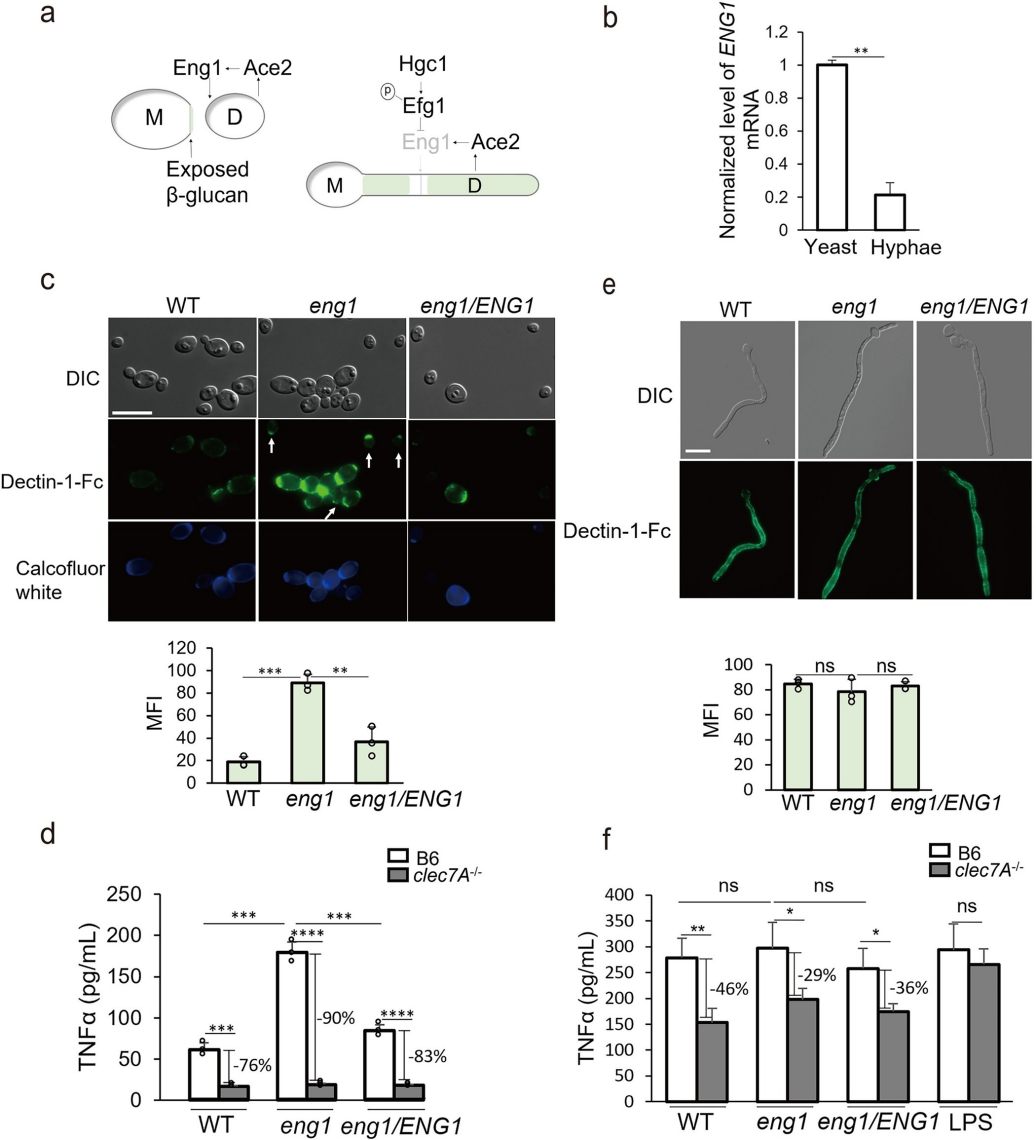
Eng1 reduces β-glucan exposure in the yeast form of *C albicans*. (a) Depicted regulation of *ENG1* expression in yeast and hyphae. (b) Expression levels of *ENG1* mRNA in yeast or hyphae grown in YPD for 1 h, quantitated by qPCR. *ENG1* transcript level was normalized with *ACT1* transcript level. (c) Representative images of *C*. *albicans* cells stained with Dectin-1-Fc and secondary antibody conjugated to FITC. Yeast cells were cultured in YPD for 6 hours. The scale bar represents 10μM. Mean fluorescence intensities per area were quantitated by ImageJ. (d) The levels of TNFα in the supernatant of BMDM stimulated with fixed *C*. *albicans* yeast form by a MOI of 1:3. (e) Representative images of hyphae stained with Dectin-1-Fc and secondary antibody conjugated to FITC. Hyphae were induced in RPMI for 5 hours. Mean fluorescence intensities were quantitated by ImageJ. The scale bar represents 10μM. (f) The levels of TNFα in the supernatant of BMDM stimulated with WT, *eng1* or *eng1/ENG1* live yeast by a MOI of 1:1. Experiments were repeated at least three times. *p* Values were calculated using ANOVA with Tukey post hoc analysis (****p<0.0001; ***p<0.001, **p<0.01, *p<0.05).

The higher β-glucan exposure in hyphae relative to yeast could be attributed to a faster growth of hyphal cell wall, or a thinner outer layer of mannan [29]. It is also possible that there are yeast-specific mechanisms of β-glucan protection. In *Histoplasma capsulatum*, an endo-1,3-β-glucanase (Eng1) digests exposed β-glucan on the cell wall through the mechanism of epitope shaving [37]. *C*. *albicans* has an Eng1 ortholog [38] with endo-glucanase activity [38]. Transcription of *ENG1* is regulated by Ace2 In *C*. *albicans* [39]. Ace2 is expressed in daughter cells after cytokinesis for septum destruction [40,41]. At early G1 phase in *Sachromyces cerevisiae*, *ENG1*, as other Ace2 target genes, is expressed and secreted from the daughter cell to degrade β-glucan in the septum, leaving a bud scar on the mother side [40]. Similar regulation is expected for *C*. *albicans* yeast cells (Fig 2A). Different from yeast cells, Ace2 target genes are not expressed in germ tubes during hyphal initiation (first cell cycle) and are down-regulated during hyphal development through hypha-specific Cdk1-Hgc1 phosphorylation of Efg1, leading to cell chain formation [41], as depicted in Fig 2A. As expected for an Ace2 regulated gene, we show that *ENG1* expression is repressed during hyphal initiation (Fig 2B).

To determine if Eng1 is involved in trimming exposed β-glucan on the cell surface, we constructed an *eng1* deletion strain and an *eng1/ENG1* strain which complemented both alleles of the deleted *ENG1* by CRISPR/Cas9 [42]. The *eng1* mutant showed an increase in cell chain formation and enhanced Dectin-1 binding at septa (Fig 2C, upper panel). Dectin-1 binding of *eng1* yeast was 3-fold higher than WT yeast (Fig 2C, lower panel), and was detectable in both mother and daughter cells, which lacked detectable chitin staining (white arrows). In contrast, Dectin-1 binding in WT and *eng1/ENG1* cells was only seen at the bud scar of mother cells, but not daughter cells. The *eng1* mutant specifically showed higher exposure of β-glucan but not mannan or chitin, since ConA and WGA staining did not show differences between WT and *eng1* yeast (S2 Fig). Consistent with their increased Dectin-1 recognition, fixed *eng1* yeast cells induced significantly higher levels of TNFα in bone marrow-derived macrophages (BMDM) compared to the fixed yeast of WT or *eng1/ENG1* strains (Fig 2D). In addition, cytokine production in response to fixed yeast cells was Dectin-1-dependent. Therefore, Eng1 plays a major role in controlling β-glucan exposure in yeast, and the immune recognition of yeast cells is Dectin-1-dependent.

Hyphae of the WT, *eng1* and *eng1/ENG1* cells, grown in RPMI for 5 h, all showed strong and similar levels of Dectin-1-Fc binding along parallel cell wall (Fig 2E). Same strong Dectin-1 binding was observed in hyphae of the WT and *eng1*, grown in N-acetylglucosamine for 5 h (S3 Fig). This is consistent with the repressed *ENG1* expression in hyphae. Interestingly, mature hyphae of both the WT and *eng1* mutant lack Dectin-1 binding (S3 Fig). To examine immune activation by hyphae, we infected BMDMs with live yeast cells, which rapidly developed into hyphae during incubation. Because of the nonsynchronous nature of hyphal development, both yeast and hyphae were present during the infection. Infected as live yeast, WT, *eng1* and *eng1/ENG1* cells induced similar levels of TNFα by BMDMs (Fig 2F). Although Dectin-1^-/-^ BMDMs secreted significantly less TNFα than WT BMDMs, a large portion of TNFα production was Dectin-1 independent (Fig 2F). This suggests that other receptors, such as CR3 and EphA2 that recognize β-glucan, and Dectin-2 that recognizes mannan may mediate immune activation and cytokine production when induced by live hyphae [34,43–45]. Therefore, consistent with its reduced expression in hyphae, Eng1 plays a limited role in immune activation by hyphae under the assay condition.

### Co-regulation of β-glucan protection in yeast by Eng1 and the Yeast Wall Protein Ywp1

**Fig 3:**
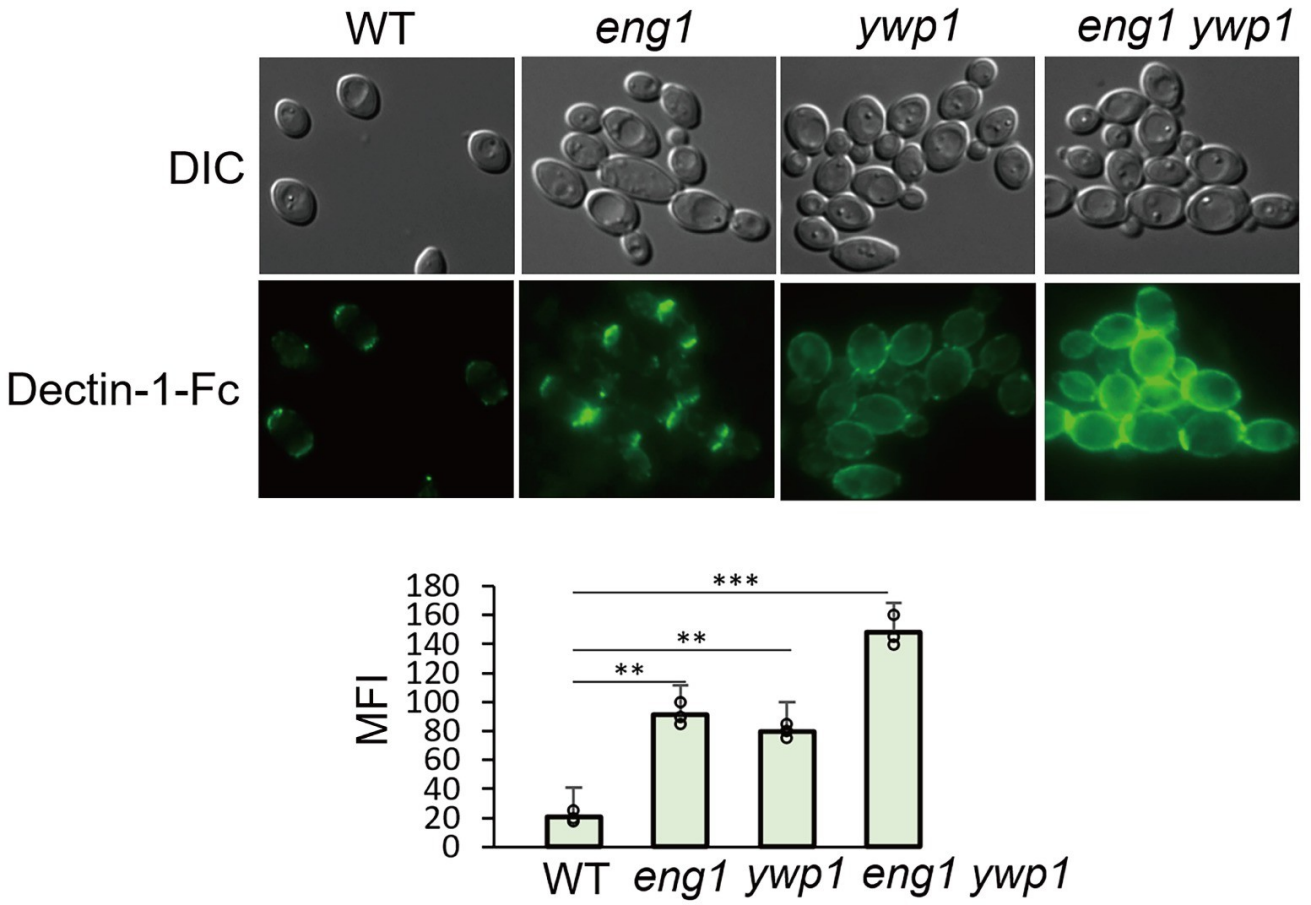
Regulation of β-glucan exposure in yeast by Eng1 and Ywp1. Representative images of *C*. *albicans* yeast cells stained with Dectin-1-Fc and secondary antibody conjugated to FITC. The scale bar represents 10μM. Mean fluorescence intensities per area were quantitated by ImageJ. *p* Values were calculated using ANOVA with Tukey post hoc analysis (***p<0.001, **p<0.01).

The *eng1* mutant showed enhanced Dectin-1 binding only at septa (Fig 2C), indicating the existence of an additional yeast-specific mechanism for β-glucan masking of the yeast cell wall outside of the septa region. The Yeast Wall Protein 1 (Ywp1), an anti-adhesin that is expressed highly in yeast cells, is implicated in blocking the accessibility to both anti-Ywp1 and anti-β-glucan antibodies to the yeast surface [34]. Using CRISPR/Cas9, we constructed an *ywp1* single deletion mutant and an *eng1 ywp1* double deletion mutant. The *ywp1* mutant showed slightly increased punctate Dectin-1 binding to the overall cell wall and tended to form aggregates (Fig 3). In comparison, the *eng1 ywp1* double mutant showed strong Dectin-1 binding to the septa and over the entire surface of the yeast cells (Fig 3). The signal in the *eng1 ywp1* double mutant was specific to Dectin-1 recognition of β-glucan, as adding laminarin or secondary antibody only did not give detectable signal on the double mutant (S1B Fig). Dectin-1 binding to the double mutant revealed the function of Ywp1 in β-glucan masking in the absence of the Eng1 glucanase. Similarly, the β-glucan trimming activity of Eng1 on the entire cell wall was better revealed in the absence of Ywp1 masking. Our finding of parallel regulation of β-glucan protection by two highly expressed yeast proteins directly links the dimorphic regulation of β-glucan exposure to the hyphal transcriptional programs [41,46].

### *ENG1* expression in yeast cells is highly regulated and is responsible for increased β-glucan exposure and immune activation of antifungal treated cells

**Fig 4:**
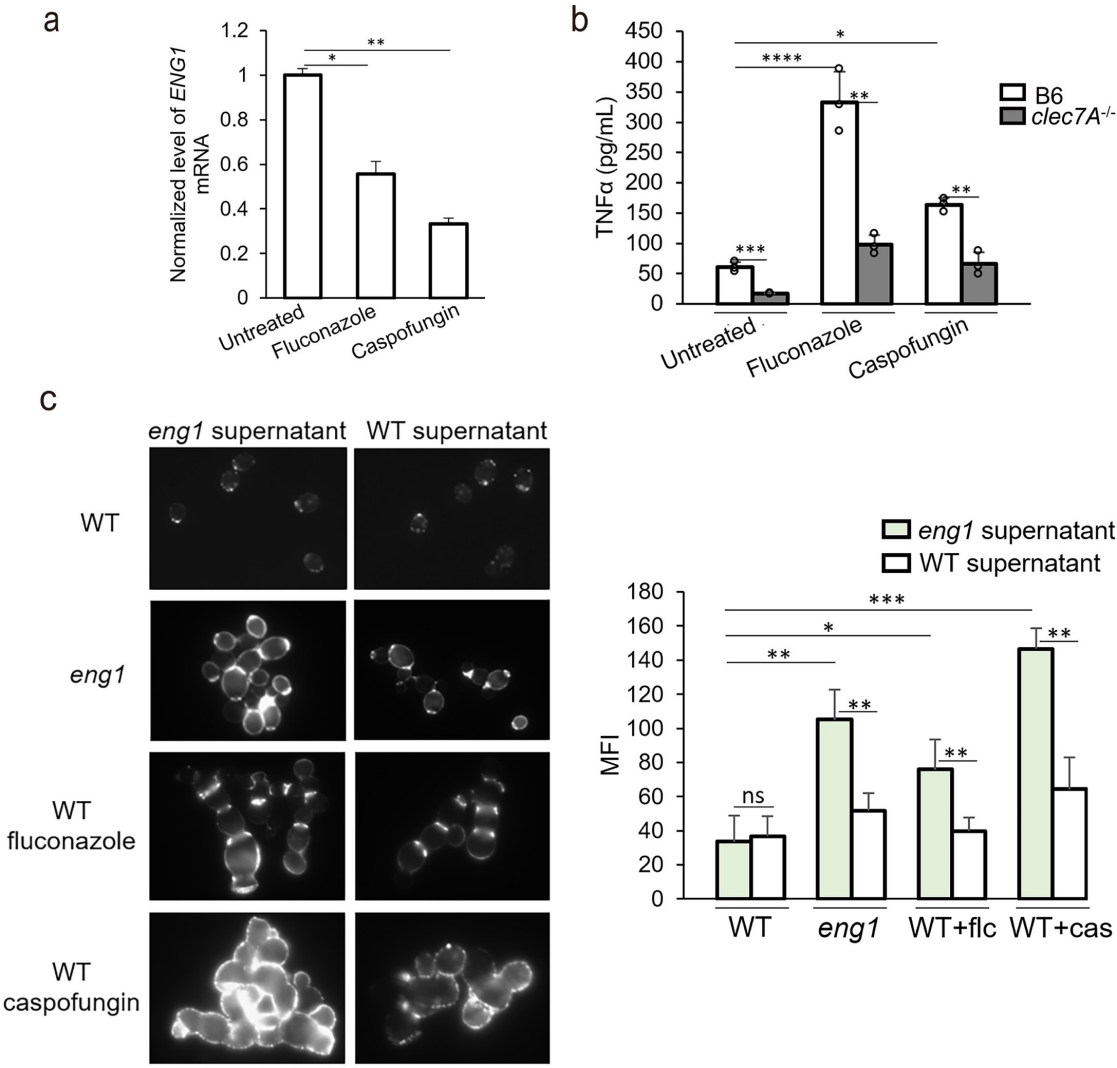
Down-regulation of *ENG1* during cell chain formation is associated with increased β-glucan exposure (a) Transcript levels of *ENG1* in fluconazole/caspofungin-treated or untreated cells. (b) The levels of TNFα in the supernatant of BMDM stimulated with fixed yeast cells by a MOI of 1:3. Cells were cultured in YPD with 10ug/mL fluconazole for overnight or 0.06ug/mL Caspofungin for 3 hours. (c) Representative images of yeast form cells stained with Dectin-1-Fc and secondary antibody conjugated to FITC. WT and *eng1* supernatant were collected and filtered from saturated overnight culture of WT or *eng1* yeast cells. Mean fluorescence intensities per area were quantitated by ImageJ. *p* Values were calculated using ANOVA with Tukey post hoc analysis (****p<0.0001; *** *p*<0.001, ** *p*<0.01, * *p*<0.05).

We also investigated whether *ENG1* expression is altered in response to treatments and conditions known to affect cell separation as *ENG1* is a target of Ace2. The anti-fungal drugs fluconazole and caspofungin have been shown to increase cell chain formation, indicating a repression of Ace2-target genes [16,47]. Indeed, *ENG1* transcript levels were lower in fluconazole or caspofungin treated cells (Fig 4A). We found that the drug-treated cells induced higher levels of TNFα in BMDMs in a Dectin-1-dependent manner (Fig 4B), indicating an increase in Dectin-1 recognition. To determine if the higher Dectin-1 recognition is due to the downregulation of Eng1, fixed WT, *eng1* and drug treated WT yeast cells were incubated overnight with the supernatant of WT culture, which should contain the secreted Eng1 enzyme. The supernatant from *eng1* cells was used as a no-Eng1 control. The *eng1* cells treated with WT supernatant had reduced Dectin-1-Fc binding as compared to the cells treated with *eng1* supernatant (Fig 4C). By contrast, the WT supernatant had no effect on WT cells. These results confirmed the β-glucan trimming ability of the Eng1 enzyme in the WT supernatant, even though it was not able to completely remove β-glucan at the septum. When the cells were treated with fluconazole or caspofungin and incubated with the *eng1* supernatant, they bound more Dectin-1-Fc than the WT cells (Fig 4C). Fluconazole-treated yeast cells had enhanced Dectin-1 binding at the septa between mother and daughter cells, similar to the *eng1* mutant. Caspofungin-treated yeast cells also had an increase in overall Dectin-1-Fc binding. When the cells were treated with either antifungal agent, incubation with WT supernatant largely reduced the Dectin-1 binding, indicating that supplemental Eng1 enzyme was able to remove exposed β-glucan on the cell wall (Fig 4C). These results suggest that reduced Eng1 expression contributes to the increased β-glucan exposure when *C*. *albicans* cells are exposed to fluconazole or caspofungin.

### Carbon sources regulate β-glucan protection through Eng1

**Fig 5:**
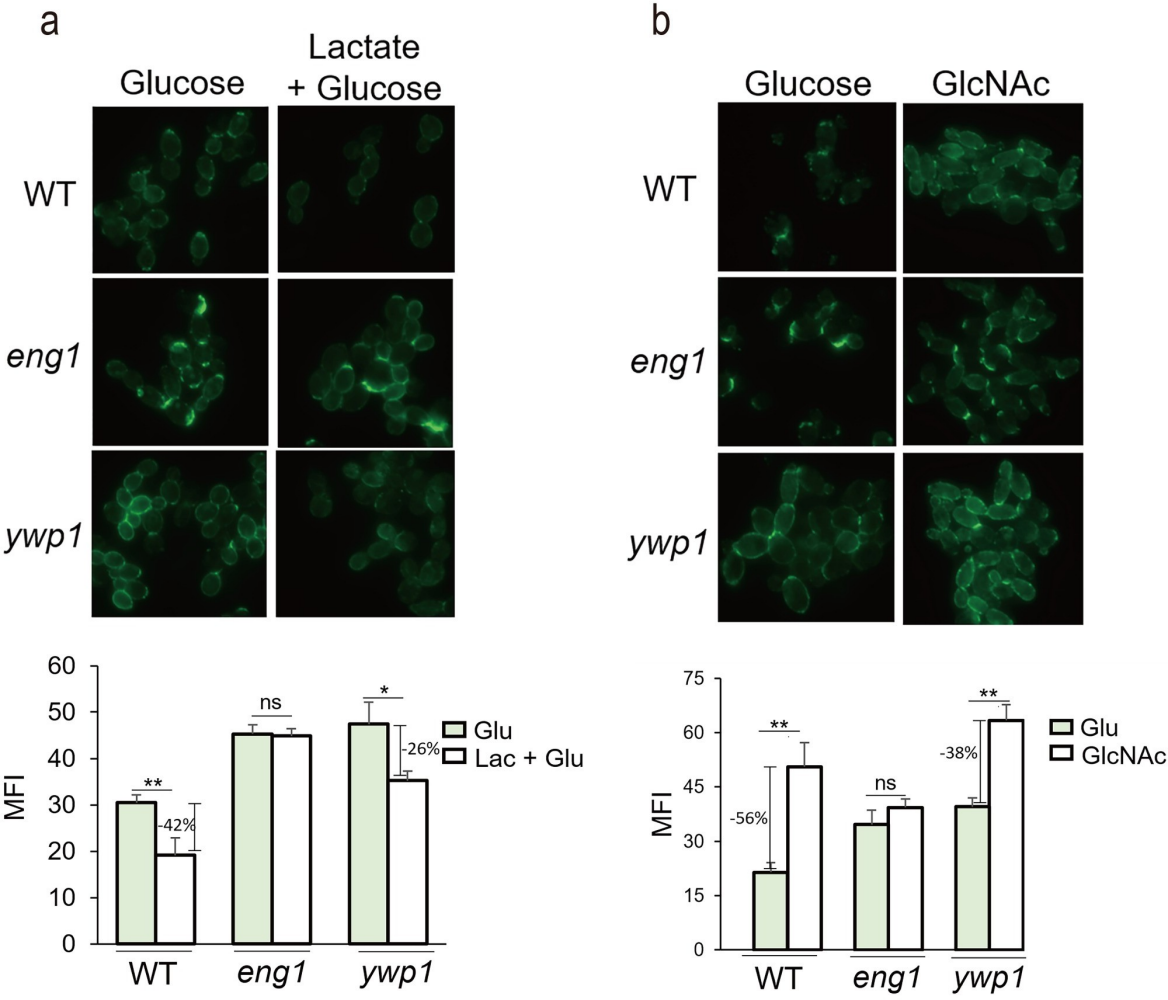
Carbon sources regulated β-glucan exposure through Eng1. (a) Representative images of *C*. *albicans* yeast cells stained with Dectin-1-Fc and secondary antibody conjugated to FITC. Cells from the overnight cultures were diluted to fresh medium containing 2% glucose alone or 1% lactate plus 1% glucose as sole carbon sources, and grown for a further 5 hours. (b) Representative images of *C*. *albicans* yeast cells stained with Dectin-1-Fc and secondary antibody conjugated to FITC. Cells were first cultured to log phase to avoid hyphae induction in the later GlcNAc culture. Then cells were transferred to fresh medium containing 2% glucose or GlcNAc as sole carbon sources, and grown for a further 5 hours. Experiment was repeated three times. Fluorescence intensities per area were quantitated by ImageJ. *p* Values were calculated using ANOVA with Tukey post hoc analysis (** *p*<0.01, * *p*<0.05).

Independently of yeast-hypha regulation, culture conditions such as alternative carbon sources are reported to regulate β-glucan protection [10,12]. Among them, lactate had been shown to induce β-glucan masking [10]. *ENG1* is upregulated in lactate containing media [21]. To evaluate the role of Eng1 in lactate-induced β-glucan protection, we cultured WT, *eng1* and *ywp1* strains in medium containing glucose alone or lactate plus glucose and examined Dectin-1 binding. While lactate induced a significantly lower level of Dectin-1 binding in the WT strains as reported, the *eng1* mutant cells did not show a reduction in Dectin-1 binding when grown in lactate compared to glucose alone (Fig 5A). In contrast, the *ywp1* mutant had significant decrease in Dectin-1-Fc binding after lactate incubation, but the level of binding to this mutant was still greater than the WT strain (Fig 5A). These results indicated that lactate-induced β-glucan masking is mediated mainly through Eng1 and that Ywp1 makes a minor contribution to this process.

N-acetylglucosamine (GlcNAc) has been implicated in increasing β-glucan exposure due to Cek1 activation [20,48]. Interestingly, Eng1 is downregulated after GlcNAc treatment [49]. To examine if Eng1 is involved in β-glucan protection in response to GlcNAc, we cultured WT, *eng1* and *ywp1* yeast cells in media containing glucose or GlcNAc. WT cells in GlcNAc showed an increase in Dectin-1 binding compared to the cells cultured in glucose (Fig 5B). In contrast, GlcNAc did not induce a higher level of Dectin-1 binding in the *eng1* mutant (Fig 5B). The *ywp1* mutant showed an increased level in Dectin-1 binding in GlcNAc than in glucose (Fig 5B). These results indicated that the GlcNAc-induced increase in β-glucan exposure is mediated mainly by Eng1.

### The *eng1* mutant modulates virulence in mice in a Dectin-1-dependent manner

**Fig 6:**
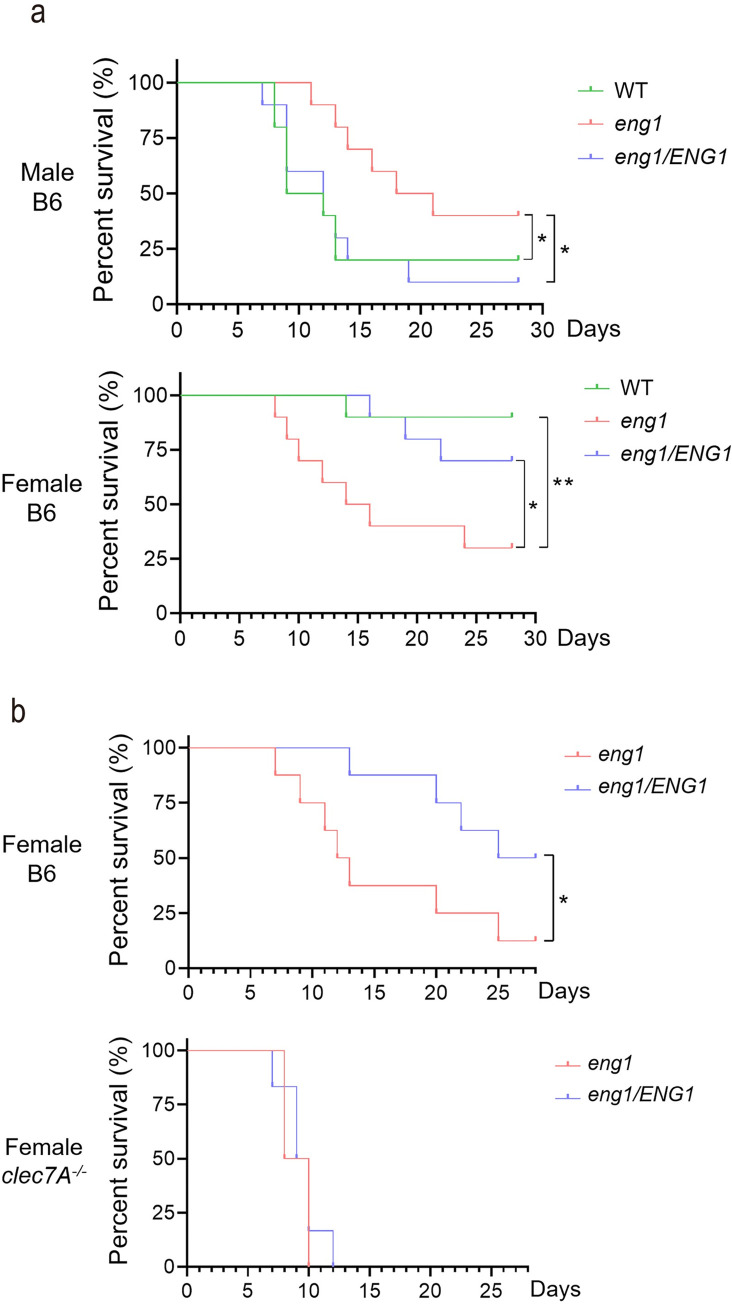
The *eng1* mutant was hypo-virulent in male and hyper-virulent in female. (a) Survival of C57BL/6 male and female mice after intravenous inoculation with 1 × 10^5^ yeast phase cells of the indicated strains of *C*. *albicans*. Experiments were done twice; n = 5 for each time. (b) Survival of C57BL/6 and Dectin-1^-/-^ female mice after intravenous inoculation with 1 × 10^5^ yeast phase cells of the indicated strains of *C*. *albicans*. Experiments were done twice; n = 3 for each time. *p* Values were calculated with Gehan-Breslow-Wilcoxon (**p<0.01; * *p*<0.05).

Eng1 is highly expressed during systemic infection [50]. To evaluate the role of Eng1 during infection, we used the well-established model of hematogenously disseminated candidiasis. Male and female C57BL/6J mice were infected with 1 x 10^5^ cells of the WT (SC5314), *eng1* deletion, or eng1/*ENG1* complemented strain by tail vein injection and monitored for survival. In male mice, the *eng1* mutant showed attenuated virulence. The median survival of male mice infected with the WT or the *ENG1* complemented strain was 10.5–12 days, whereas the median survival of male mice infected with the *eng1* cells was 19.5 days (Fig 6A, top panel). The attenuated virulence by mutants with increased β-glucan exposure is consistent with the immune protective role of Dectin-1 in systemic Candidiasis [6]. Consistent with the immune protective role of Dectin-1, the attenuated virulence of the *eng1* mutant is dependent on Dectin-1 (S4 Fig). Female mice were less susceptible than male mice to infection with the WT strain SC5314 (Fig 6A), as reported [51]. Unexpectedly, the *eng1* mutant was hyper-virulent in female mice. The median survival of female mice infected with the WT or *ENG1* complemented strain was more than 28 days whereas the median survival of mice infected with the *eng1* mutant yeast was 15 days (Fig 6A, bottom panel). To determine if the hyper-virulent effect of the *eng1* mutant was Dectin-1-dependent, female C57BL/6J mice and female Dectin-1 deficient *clec7A*^*-/-*^ mice were infected with 1 x 10^5^ cells of the *eng1* deletion or the *eng1*/*ENG1* complemented strain. The hyper-virulence effect of the *eng1* mutant was reproduced in the female C57BL/6J mice (Fig 6B). In contrast, the Dectin-1-deficient female *clec7A*^*-/-*^ mice were highly susceptible to the *eng1/ENG1* complemented strain in comparison to C57BL/6J mice, consistent with the immune protective role of Dectin-1 (Fig 6B). Furthermore, *clec7A*^*-/-*^ mice showed similar susceptibility to the *eng1* deletion and *eng1/ENG1* complemented strain, and all died within 10–11 days (Fig 6B).

### The *eng1* mutant activates a greater renal immune response

**Fig 7:**
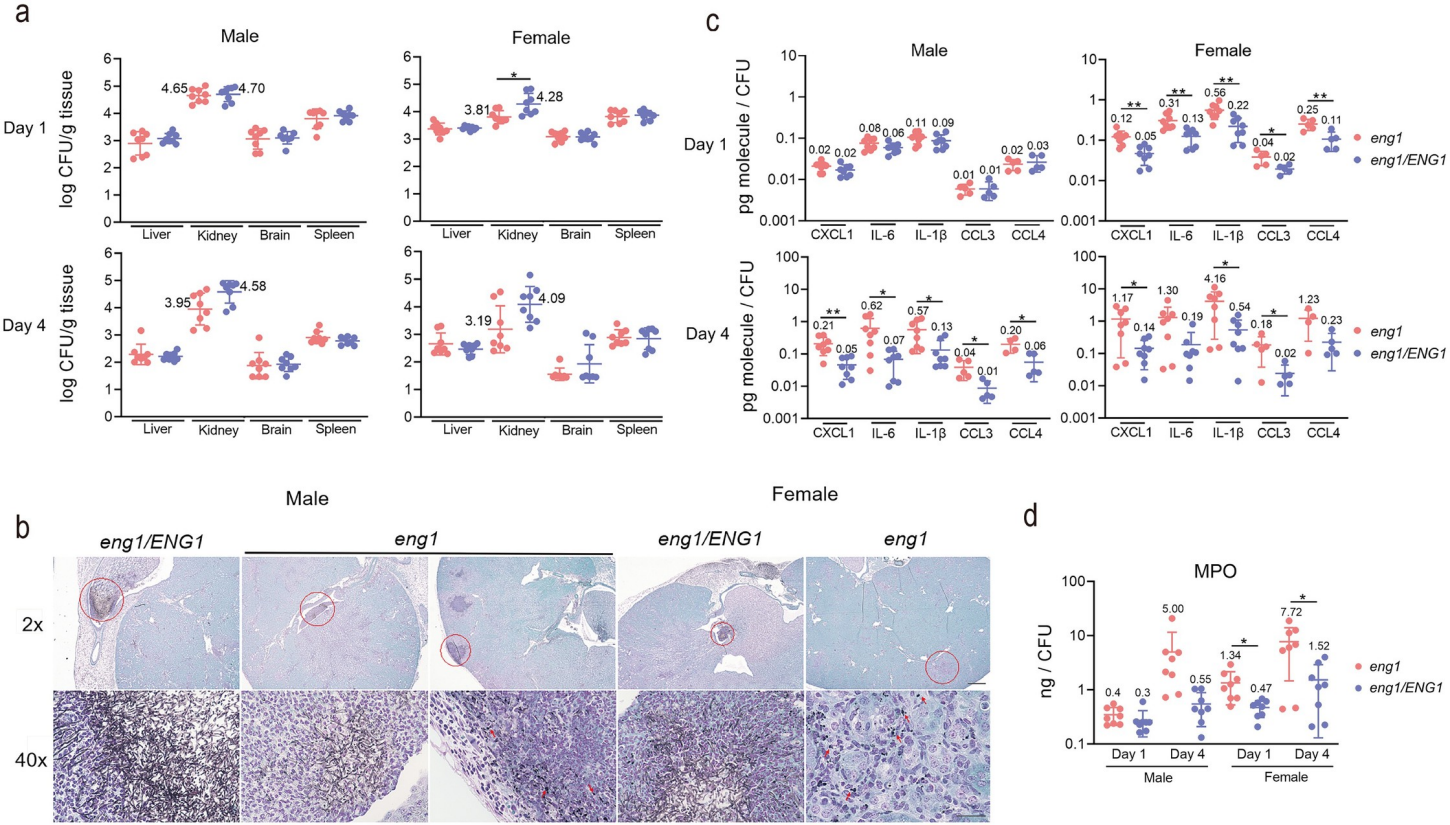
(a) Fungal burden of the kidney, brain, spleen, and liver of mice at 1-day and 4-day postinfection after inoculation with 1 × 10^5^ *eng1* deletion mutant or *eng1/ENG1* complemented *C*. *albicans* yeast. Results are median ± interquartile range with 8 mice per strain. Experiments were done twice; n = 3 for the first time and n = 5 for the second time. *p* Values were calculated with Mann-Whitney test (*p<0.05). (b) Representative images of kidney histology slides of PAS-stained paraffin sections from infected kidneys at day 4 post infection. Sections from two sets of kidneys were analyzed. Circled area in 2x image is shown in the 40x image bellow. Scale bars in 2x and 40x are 500 μM and 25 μM, respectively. Arrows indicate yeast-like cells. (c) Relative cytokine/chemokine levels at 1-day and 4-day post-infection. (d) Relative MPO levels at 1-day and 4-day post-infection. *p* Values were calculated with Unpaired t-test (**p<0.01; * *p*<0.05).

To determine why the *eng1* mutant had different virulence in male vs. female mice, we analyzed the organ fungal burden and inflammatory response of mice after 1 and 4 days of infection. We found that the fungal burden in the liver, kidney, brain, and spleen of male mice infected with the *eng1* mutant was similar to that of mice infected with the *ENG1* complemented strain at both time points (Fig 7A, left panels). The fungal burden of the kidneys of the female mice infected with the *eng1* mutant was lower at day 1 relative to mice infected with the *ENG1* complemented strain. There was a similar trend at day 4, but the difference was not statistically significant (Fig 7A, right panels). Histopathological analysis shows that the *ENG1* complemented cells are located in the pelvis region as clusters of mostly hyphae in both male and female kidneys at day 4 post infection (Fig 7B). In male mice, the *eng1* cells are found in the renal pelvis in clusters of mostly hyphae; they are also found in yeast and hyphal forms scattered in the renal cortex region (Fig 7B). We are unable to find the *eng1* cells in the pelvis region in female kidneys, but observed some yeast-looking *eng1* cells dispersed in renal cortex (Fig 7B). Scattered yeast cells in renal cortex are harder to identify than clusters of hyphae in the renal cortex. Overall, the amounts of fungal cells observed in kidney pathology slides correlate with the renal fungal burdens at day 4 post infection (Fig 7A).

To determine if the *eng1* mutant elicited a different inflammatory response than the *ENG1* complemented strain, we analyzed the levels of cytokines and chemokines that are upregulated in the kidney during systemic candidiasis [4]. We found the levels of CXCL1, IL-6, IL-1β, CCL3 and CCL4 were higher in the kidney homogenates of female mice infected with the *eng1* strain at days 1 and 4 post-infection in comparison to that infected with the *ENG1* strain (Figs 7C and S5). Because Pearson correlation analysis indicates that cytokine levels in each kidney are highly correlated with the corresponding fungal burden (S6 Fig), the data were normalized to the kidney fungal burden of the individual mice [52]. After 1 day of infection in the male mice, the chemokines and cytokines levels were similar in animals infected with the *eng1* mutant and the *ENG1* complemented strain (Fig 7C upper left panel). After 4 days infection, the levels of these inflammatory mediators in both male and female mice infected with the *eng1* mutant were significantly higher than those in mice infected with the *ENG1* complemented strain (Fig 7C lower left panel). In the female mice, the levels of chemokines and cytokines in the kidneys were consistently higher and induced earlier than in the male mice (Fig 7C), indicating that female mice mounted a stronger inflammatory response to *C*. *albicans*. Collectively, these results indicate that the *eng1* mutant induces a greater inflammatory response in the kidneys and that female mice mount a stronger inflammatory response to *C*. *albicans* than male mice.

We also measured the levels of myeloperoxidase (MPO) to assess the relative amounts of phagocytes (neutrophils, monocytes, and macrophages) [53–55] in the kidneys. In male mice, the *eng1-*infected kidneys did not show significantly higher levels of MPO compared to *ENG1* infected kidneys on both day 1 and day 4 (Fig 7D). In female mice, the *eng1* infected kidneys showed significantly higher levels of MPO starting on day 1, and the increase sustained to day 4 (Fig 7D). The higher MPO levels indicated earlier and greater phagocyte accumulation in the *eng1* infected female kidneys. These results coincided with the lower survival rates of *eng1* infected female mice.

## Discussion

### β-glucan is masked in yeast by Eng1 and Ywp1

Whether and how β-glucan protection is differentially regulated during hyphal morphogenesis is not fully understood. Here we show that the endo-1,3-glucanase Eng1 is expressed higher in yeast than in hyphae, and together with Ywp1, controls β-glucan exposure in yeast cells. An *eng1* deletion mutant shows enhanced Dectin-1 binding at the septa of yeast cells, while an *eng1 ywp1* double mutant shows strong punctate Dectin-1 binding on the entire cell surface. Similar Dectin-1 binding is also observed on germ-tubes during hyphal initiation. Our data suggest that repression of *ENG1* and *YWP1* expression during hyphal initiation leads to β-glucan exposure on hyphae. *YWP1* is specifically expressed in yeast cells, not in hyphae [56]. During hyphal development, phosphorylation of the Efg1 transcription factor by hypha-specific Cdk1-Hgc1 represses Ace2-regulated genes (including *ENG1*), leading to the formation of cell chains [41]. Thus, our findings provide a direct link between β-glucan exposure and the hyphal transcriptional programs. Interestingly, immune activation by yeast cells is Dectin-1-dependent, but a large portion of TNFα production in response to hyphae is Dectin-1 independent despite the high level of β-glucan in hyphae. Other receptors such as CR3 and EphA2 that recognize β-glucan, and additional cell wall PAMPs (e.g. mannan epitopes) may also contribute to innate immune recognition of *C*. *albicans* hyphae and mediate immune activation [34,43–45]. Consistent with our data, Dectin-1 is required for Syk activation by yeast cells while Dectin-2 is required for Syk activation by hyphae [43,57]. The differential Dectin-1 dependence between yeast and hyphae could be due to cell sizes and different PAMPs on yeast and hyphal wall.

We show that in addition to being regulated during yeast to hyphal transitions, *ENG1* transcription and β-glucan exposure are also regulated by lactate, GlcNAc, and increased synthesis caused by cell membrane/wall perturbations by antifungals. It has been reported that Cek1 hyper-activation increases β-glucan exposure [20]. This is probably caused by reduced levels of Eng1, because RNA-seq data indicate that *ENG1* transcription is repressed by the Cek1 pathway [58]. We expect that *ENG1* expression is dynamically regulated in response to additional environmental cues that affect cell separation and β-glucan exposure.

Similar to HcEng1 in *H*. *capsulatum* [37], the *C*. *albicans* Eng1 reduces β-glucan exposure in yeast likely by triming excess β-glucan as an endo-1,3-glucanase [38]. In *H*. *capsulatum*, β-glucan is also masked by an outer layer of α-glucan [59]. In *C*. *albicans*, Ywp1 contributes to β-glucan masking in yeast cells [34] The *eng1 ywp1* yeast cells show strong punctate Dectin-1 binding on the entire cell surface. Thus, Eng1 not only acts at the site of septa but also over the entire cell surface when β-glucan is not masked by Ywp1. Ywp1 is not required for β-glucan masking at the site of septa as the *eng1* single mutant showed strong Dectin-1 binding at septa. Thus, we propose that Eng1-mediated β-glucan trimming and Ywp1-mediated β-glucan masking, are two parallel mechanisms utilized by *C*. *albicans* to reduce β-glucan exposure in yeast. The combination of β-glucan masking and trimming maybe a general mechanism for control β-glucan exposure in fungi [37].

### The *eng1* mutant induces a greater immune response and modulates virulence via Dectin-1

Pirofski and Casadevall [60] postulate that tissue damage can be caused by both the microbial pathogen and the host inflammatory response. In the current study, the kidney fungal burden of mice infected with the *eng1* mutant was generally similar to that of mice infected with the *ENG1* complemented strain, even though the survival was different. Interestingly, the *eng1* cells were found in both yeast and hyphal forms in infected male kidneys and mostly as yeast cells in female kidneys at day 4 post infection. The *ENG1* complemented cells were found mostly in hyphal form as expected for WT *C*. *albicans*. However, the *eng1* mutant was not found to impair cell growth or hyphal development under *in vitro* conditions. Therefore, the yeast form of the *eng1* mutant in infected female kidneys is likely due to the increased β-glucan exposure of the *eng1* mutant that triggered a stronger host immune response during infection. Our data indicate that altered mortality of mice infected with the *eng1* mutant may be caused by the host inflammatory response elicited by greater β-glucan exposure in the *eng1* mutant. However, fungal burden, kidney pathology and cytokines were analyzed early during infection. Additional experiments are needed in later time points to elucidate the cause of lethality in the *eng1* infected female mice.

Dectin-1 is known to play a protective role in defending against systemic *C*. *albicans* infection [6,61]. Our observation of attenuated virulence of the *eng1* mutant in male mice and two previous studies of mutants with increased β-glucan exposure in the systemic candidiasis model [15,58] are supportive of this role of Dectin-1. However, we found that the *eng1* mutant was hyper-virulent in female mice. Dectin-1-deficient female mice infected with either the *eng1* mutant strain or the *eng1/ENG1* complemented strain showed similarly low survival. This could mean that Dectin-1 deficient mice are hypersusceptible to all *C*. *albicans* strains regardless the levels of β-glucan exposure. However, we reason that the lethality associated with the *eng1* mutant in female mice is likely mediated through Dectin-1 because only yeast form of the *eng1* mutant showed increased β-glucan exposure, and Dectin-1 deficiency blocked immune activation by yeast cells of the WT and *eng1* mutant. This is the first report to indicate that Dectin-1 activation can be detrimental in *Candida* infection. Type I interferon has been shown to have a deleterious effect during systemic infection by *C*. *albicans* [62]. Mice lacking a functional IFN-I receptor showed an improved survival in response to *C*. *albicans* (S5314), and strikingly this increased survival was not paralleled by a lower fungal burden [62]. IFN-I signaling controls the recruitment of inflammatory monocytes and neutrophils to infected kidneys [62]. Within kidneys, monocytes differentiate into inflammatory DCs. The increased activity of inflammatory monotypes and neutrophils lead to hyper-inflammation and lethal kidney pathology later in the infection [62]. IFN-I signaling in renal dendritic cells in response to *C*. *albicans* is largely dependent on Dectin-1-mediated Syk activation [63]. The higher MPO level in the kidneys of female mice infected with the *eng1* mutant suggests the possibility that the enhanced virulence of the *eng1* mutant was due to the induction of a pathologic inflammatory response via Dectin-1.

In summary, this study demonstrates that β-glucan in yeast is masked from recognition of Dectin-1 by two highly expressed yeast proteins, the endo-1,3-β-glucanase Eng1 and the Yeast Wall Protein Ywp1. β-glucan is exposed during the yeast-to-hypha transition. The dynamic increase in β-glucan exposure and Dectin-1-mediated immune activation during germ-tube formation could be a critical determinate for the outcome of a *C*. *albicans* infection. Regulating the level of β-glucan exposure in yeast is important for modulating the balance between immune protection and immunopathogenesis.

## Material and methods

### Ethics statement

All animal studies were approved by the Institutional Animal Care and Use Committee (IACUC) of the Los Angeles Biomedical Research Institute and UC Irvine.

### Source of mice

C57BL/6 mice (6–10 weeks old) were from The Jackson Laboratory (Bar Harbor, ME). Dectin-1^-/-^ mice were a kind gift from Yoichiro Iwakura (University of Tokyo, Japan) bred on a BL/6 background as described [64]. Animal studies were compliant with all applicable provisions established by the Animal Welfare Act and the Public Health Services Policy on the Humane Care and Use of Laboratory Animals.

### Media and growth conditions

The strains used in this study are listed in S1 Table. Strains were grown in YEP (1% yeast extract, 2% peptone, 0.015% L-tryptophan) plus 2% dextrose, or synthetic complete medium (0.17% Difco yeast nitrogen base w/o ammonium sulfate, 0.5% ammonium sulfate, complete supplement amino acid mixture) plus 2% glucose unless otherwise described. RPMI1640 medium were used for hyphae growth. Yeast cells were cultured at 30°C. Culture for hyphae was pre-warmed and maintained at 37°C. For the carbon sources experiment, culturing conditions were adapted from an established protocol (12). Overnight *C*. *albicans* yeast culture was diluted into fresh SC medium containing glucose or lactate plus glucose as sole carbon sources to an OD_600_ of 0.2, and incubated at 30°C for 5 hours for analysis. Or overnight *C*. *albicans* yeast culture was diluted into fresh SCD medium to an OD_600_ of 0.1, and grown at 30°C for 4 hours for log phase. Then cells were spin down and washed once with PBS and added to fresh SC medium containing glucose or GlcNAc as sole carbon sources for a further 5-hour growth.

### Plasmid and *C*. *albicans* strain construction

Strains used in this study were listed in S1 Table and primers in S2 Table. Gene deletion and complementation were constructed by CRISPR/Cas9 as described [42].

BES119-*ENG1* plasmid: The *ENG1* complementation repair template was comprised of 2 pieces of PCR products generated using primer 10,11 and primer 12,13. The repair template plasmid was constructed by integrating the PCR products into the *BES119* plasmid [65] by Gibson assembly [66] for amplification in *E*. *coli*. On the day of transformation, repair template plasmids were isolated with the GeneJET Plasmid Miniprep Kit (Thermo Scientific) from the overnight *E*. *coli* culture and digested with SacI and EcoRV.

*eng1* deletion strain: *ENG1* was deleted in the SC5314 *LEU2* heterozygous knockout strain which was kindly provided by the Hernday lab [42]. Primer 1 was used to generate the sgRNA near the 5’ end of the *ENG1* open reading frame, and primer 2 was used to generate the sgRNA near the 3’ end. Primer 3 and 4 were used for the repair template, which contained the complemented sequence of the sgRNA for *ENG1* complementation. Primer 5–9 were used to confirm the deletion of the *ENG1* DNA sequence by colony PCR.

*ENG1* complemented strain: *ENG1* was complemented in the *eng1* strain. Primer 9 was used to generate the sgRNA. Repair template was isolated from the BES119-*ENG1* plasmid. Primer 14 and 15 were used in couple with primer 7 and 8 to confirm the correct insertion of the *ENG1* DNA sequence by colony PCR.

*ywp1* and *eng1 ywp1* deletion strain: *YWP1* was deleted in the SC5314 *LEU2* heterozygous knockout strain to construct *ywp1* single deletion strain, and in *eng1* to generate *eng1 ywp1* double deletion strain. Primer 18 was used to generate the sgRNA. Primer 19 and 20 were used for the repair template. Primer 21–24 were used to confirm the deletion of the *YWP1* DNA sequence by colony PCR.

### Dectin-1-Fc staining

Staining method was derived from an established protocol [67]. Soluble Fc-mDectin-1a containing the C-terminal extracellular domain of mouse Dectin-1a fused with the human IgG1 Fc domain was purchased from Invivogen. After cultured in desired conditions, 5 x 10^6^ cells were harvested, centrifuged and washed twice with PBS. After fixed with 4% formaldehyde for 15 minutes, yeast cells were washed twice with PBS and twice with binding buffer (0.5% BSA, 5mM EDTA, 2mM Sodium Azide) and incubated with 0.5 μg Dectin-1-Fc protein for 1 hour at 4 degrees. Cells were then washed twice with binding buffer and incubated with 0.5 μg secondary FITC-conjugated Rabbit anti-Human IgG for 1 hour at 4 degrees in dark. For S1 Fig, Dectin-1-Fc was pre-incubated with 1mg/mL of laminarin for 20 minutes. Or, secondary antibody was directly added to fixed samples and incubated for 1 hour. Cells were then washed three times with wash buffer and processed for microscopy. Images were obtained on a Zeiss Axioplan 2 or an inverted Zeiss Axio Observer.Z1 microscope (Carl Zeiss MicroImaging, Inc., Thornwood, NY) fluorescent system equipped with the AttoArc HBO 100 and the X-Cite series 120 mercury lamps, respectively. Both fluorescence microscopes were equipped with GFP and DAPI (4′,6′-diamidino-2-phenylindole) filter sets. Images were taken using a ×100 NA 1.4 objective lens. Processing was done using the software ImageJ (National Institutes of Health, USA), as well as Photoshop and Illustrator (Adobe Systems, Inc., Mountain View, CA). Mean fluorescence intensity per unit area was determined based on at least three randomly selected images with at least 100 cells in total by ImageJ. MFI values were calculated as the average integrated intensity of the fluorescence over the total cell area based on DIC images, then subtracted by background signal.

### WGA / ConA staining

WT and *eng1* yeast cells were cultured in YPD for 6 h, then fixed and washed as described above. Cells were incubated with 100 μg/mL Alexa Fluor 647—conjugated ConA (Sigma) in PBS for 45min in dark at RT. Then cells were washed with PBS twice, and incubated with 100 μg/mL FITC-conjugated WGA (Sigma) in PBS for 45min in dark at RT. Cells were washed three times and proceed for microscope.

### Macrophage infection and cytokine measurement

BMDM derivation was carried out as described [68]. Bone marrow cells were harvested from the femurs and tibia of C57BL/6 WT or Dectin-1^-/-^ mice. The bones were trimmed at each end and centrifuged at 800 × g for 10 s, and bone marrow cells were suspended in growth medium (Dulbecco’s modified Eagle’s medium plus10% FBS and 1% penicillin/streptomycin). Macrophages were derived by culturing bone marrow cells in growth medium plus 20ng/mL mCSF for 7 days. Medium with fresh mCSF was added every 2–3 days. Then macrophages were harvested by treating with Cell Stripper solution (Corning) for 15 minutes and scraping. Cells were counted with a hemocytometer and 10^5^ cells were plated to each well in a 96 well plate for overnight. Overnight *C*. *albicans* yeast culture were diluted 1:20 in fresh YPD medium and grew for 6 hours. Cells were then fixed with 4% formaldehyde and washed 4 times with PBS. Bone marrow-derived macrophages were stimulated with fixed yeast (MOI = 3) or live overnight yeast (MOI = 1) for 6 hours at 37 degrees with 5% CO_2_. Co-culture supernatant was collected, and cytokines were measured by commercially available Ready-Set-Go cytokine kits (eBioscience).

### Quantitative PCR

Methods for RNA isolation were carried out as previously described [69]. cDNA was synthesized from 1μg total RNA using the BioRad iScript Reverse Transcription Kit. Quantitative PCR using the BioRad SYBR Green mix and primer 16 and 17 was performed on the BioRad iCycler. Cycle conditions were 95°C for 1min, then 39 cycles of 95°C for 10s, 56°C for 45s, and 68°C for 20s. *ENG1* transcript level was normalized with *ACT1* transcript level.

### *In vivo* assays

*In vivo* assays were carried out as described [70]. Male and female C57BL/6 and Dectin-1^-/-^ mice (6–8 weeks) were inoculated via the lateral tail vein with 1x10^5^ *C*. *albicans* cells per mouse. They were monitored 3 times daily for survival. For fungal burden and cytokine measurements, the mice were sacrificed after 1 and 4 days of infection. One kidney from each mouse was harvested, weighed, and homogenized. An aliquot of the homogenate was quantitatively cultured and the remaining sample was clarified by centrifugation. The supernatant was collected and stored at -80°C. On a later date, the cytokine content of the homogenates was determined by Luminex cytometric bead array.

### Statistical analysis

At least three biological replicates were performed for all experiments, and the results are expressed as mean values ± standard deviation. Data were analyzed using student t-test, ANOVA with Tukey post hoc or Mann-Whitney test by GraphPad Prism (ver. 8.0) as indicated in the figure legends. A probability level of 5% (p < 0.05) was considered significant. Pearson correlation analysis was done by R Studio.

## Supporting information

S1 Fig Dectin-1 staining of WT hyphae, WT yeast and *eng1 ywp1* yeast using laminarin and secondary antibody only as staining controls. Hyphae was induced in YPD for 1 h at 37°C. WT and *eng1 ywp1* yeast were cultured in YPD for 6h at 30°C. Dectin-1-Fc was pre-incubated with 1mg/mL of laminarin for 20 minutes before added to fixed cells and incubated for 1 hour. Or, secondary antibody was directly added to fixed samples and incubated for 1 hour. Experiment was repeated three times.
(TIF)

S2 Fig WGA and ConA staining of WT and *eng1* yeast. Mean fluorescence intensities per area were quantitated by ImageJ. Significant analysis was calculated with Unpaired t-test. Experiment was repeated three times.
(TIF)

S3 Fig Representative images of hyphae stained with Dectin-1-Fc and secondary antibody conjugated to FITC. Hyphae were induced in SC with 2% N-acetylglucosamine for 5 hours, overnight or in RPMI overnight. The scale bar represents 10μM.
(TIF)

S4 Fig Survival of Dectin-1^-/-^ male mice after intravenous inoculation with 1× 10^5^ yeast phase cells of the indicated strains of *C*. *albicans*. Experiments were done twice; n = 3 for each time. *p* Values were calculated with Gehan-Breslow-Wilcoxon (* *p*<0.05). (TIF)

S5 Fig (a) Relative renal cytokine/chemokine levels at 1-day and 4-day post-infection. (b) Levels of renal IL-10 are bellow or similar to that of uninfected controls.
(TIF)

S6 Fig Pearson correlation analysis of cytokine/chemokine levels and fungal burdens. (TIF)

S1 Table Strains used in this study. (TIF)

S2 Table Primers used in this study. (XLSX)
