# Supplementary figures and images for: Control of β-glucan exposure by the endo-1,3-glucanase Eng1 in Candida albicans modulates virulence
Source: PLoS Pathog. 2022 Jan 7;18(1):e1010192. doi: 10.1371/journal.ppat.1010192 (PMC8775328; doi:10.1371/journal.ppat.1010192)

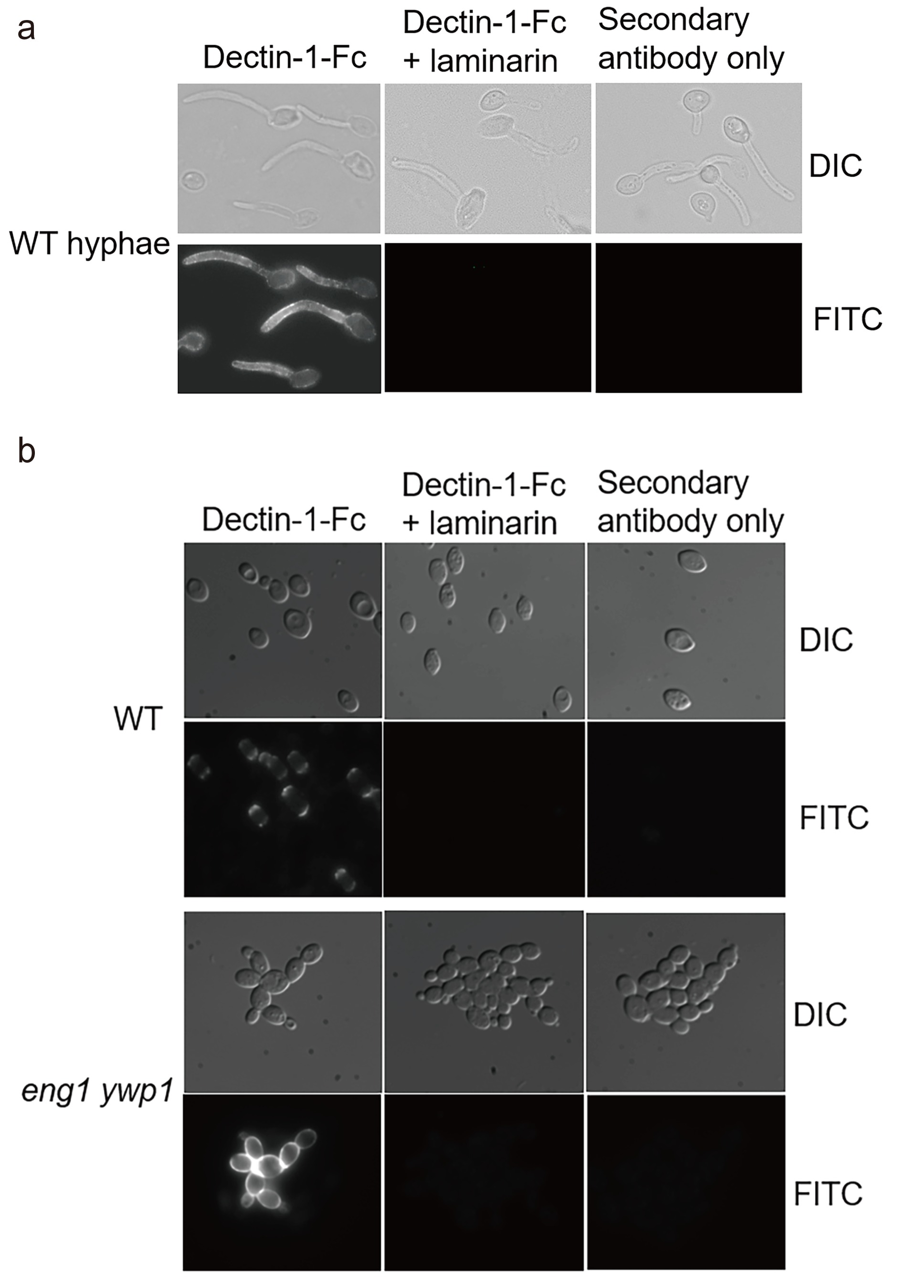

Supplement: S1 Fig — Hyphae was induced in YPD for 1 h at 37°C. WT and eng1 ywp1 yeast were cultured in YPD for 6h at 30°C. Dectin-1-Fc was pre-incubated with 1mg/mL of laminarin for 20 minutes before added to fixed cells and incubated for 1 hour. Or, secondary antibody was directly added to fixed samples and incubated for 1 hour. Experiment was repeated three times. (TIF) [file ppat.1010192.s001.tif]

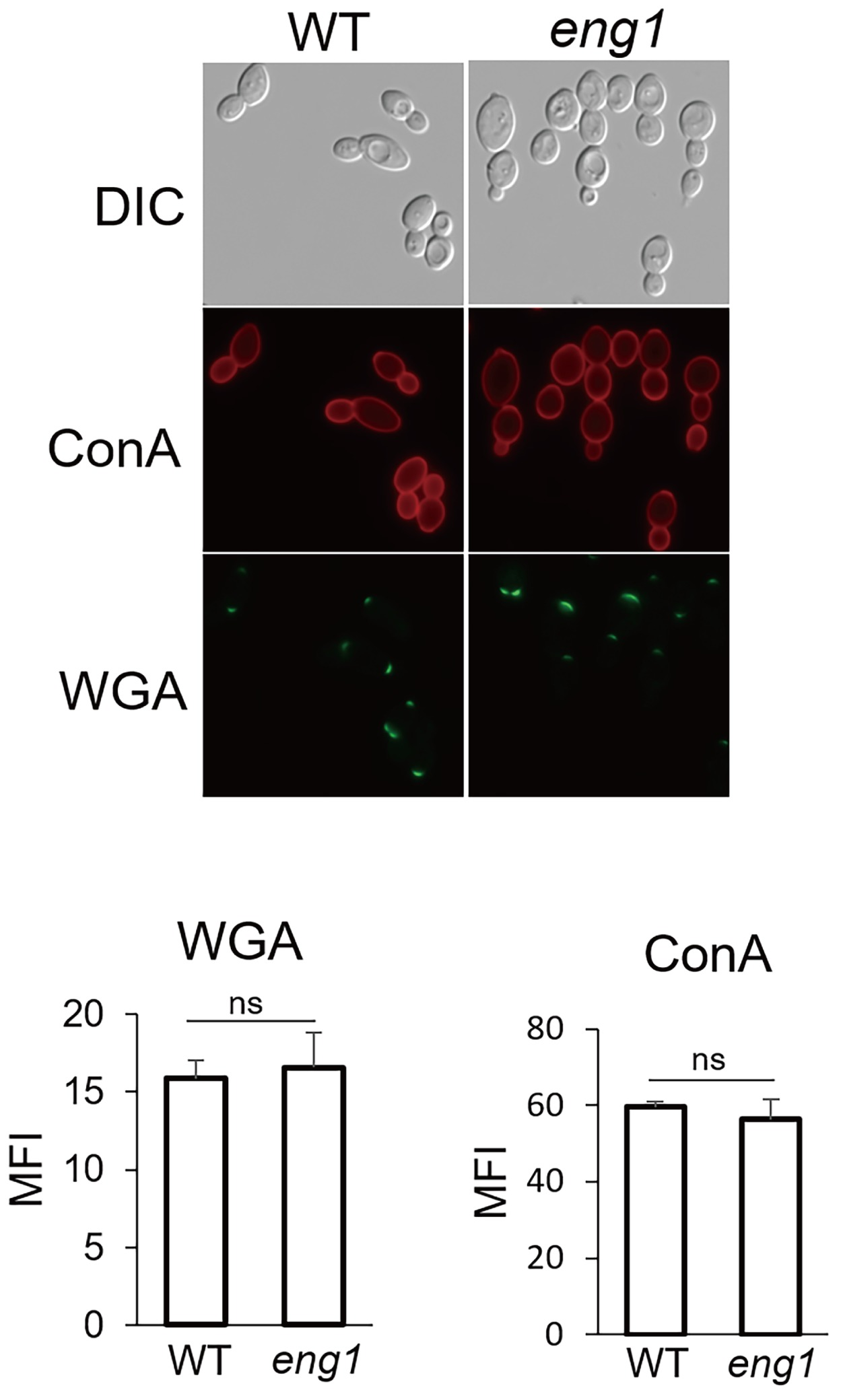

Supplement: S2 Fig — Mean fluorescence intensities per area were quantitated by ImageJ. Significant analysis was calculated with Unpaired t-test. Experiment was repeated three times. (TIF) [file ppat.1010192.s002.tif]

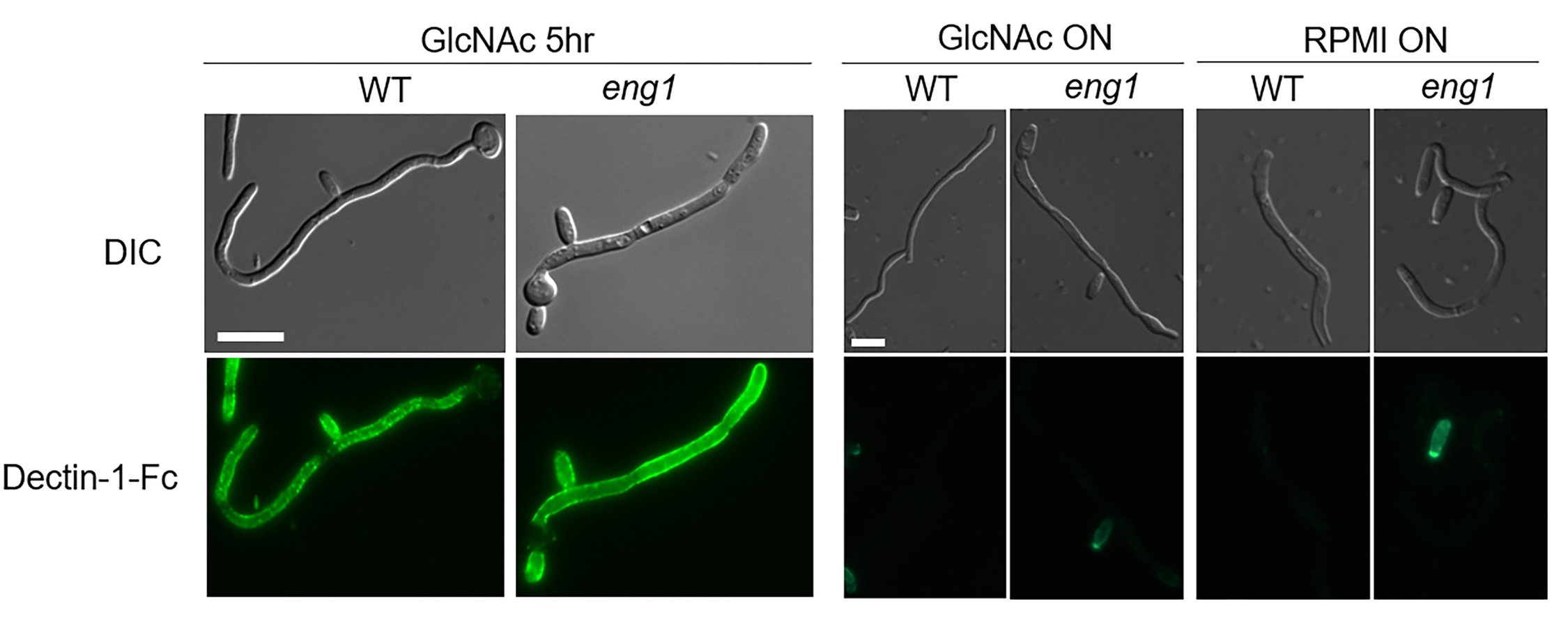

Supplement: S3 Fig — Hyphae were induced in SC with 2% N-acetylglucosamine for 5 hours, overnight or in RPMI overnight. The scale bar represents 10μM. (TIF) [file ppat.1010192.s003.tif]

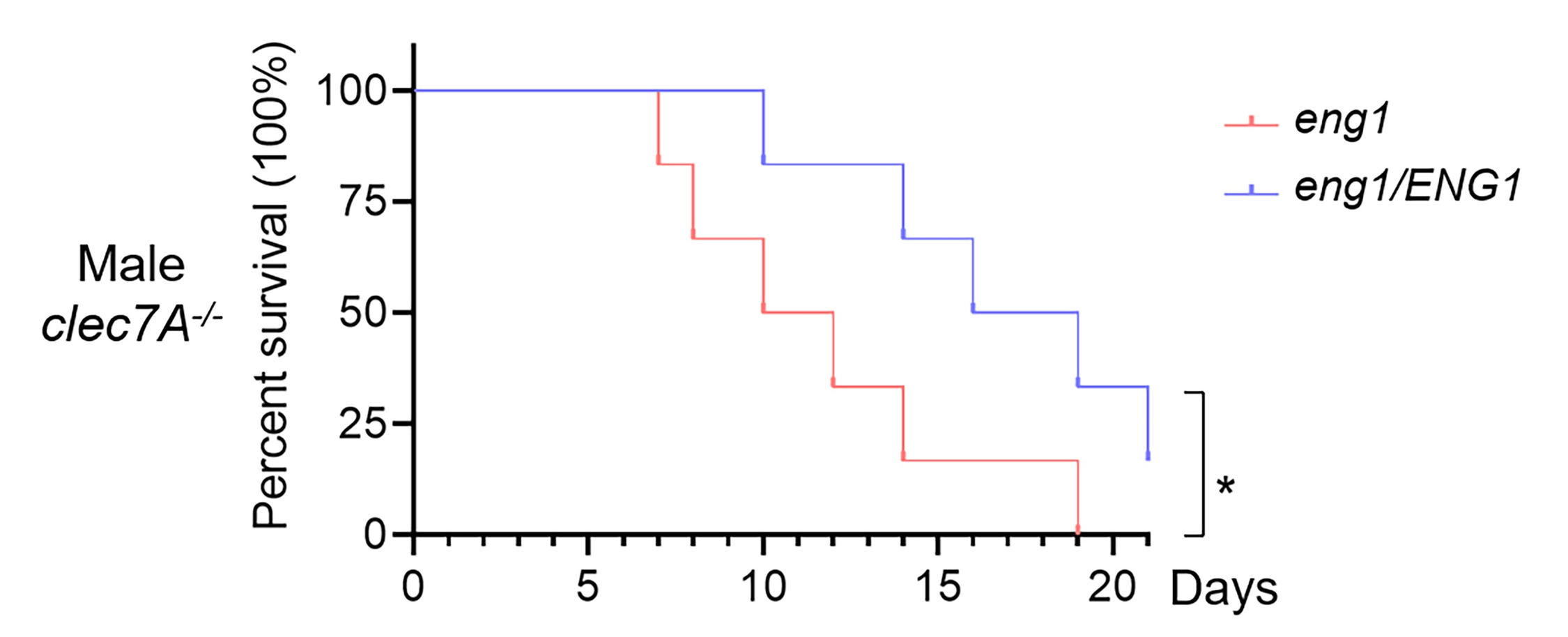

Supplement: S4 Fig — (TIF) [file ppat.1010192.s004.tif]

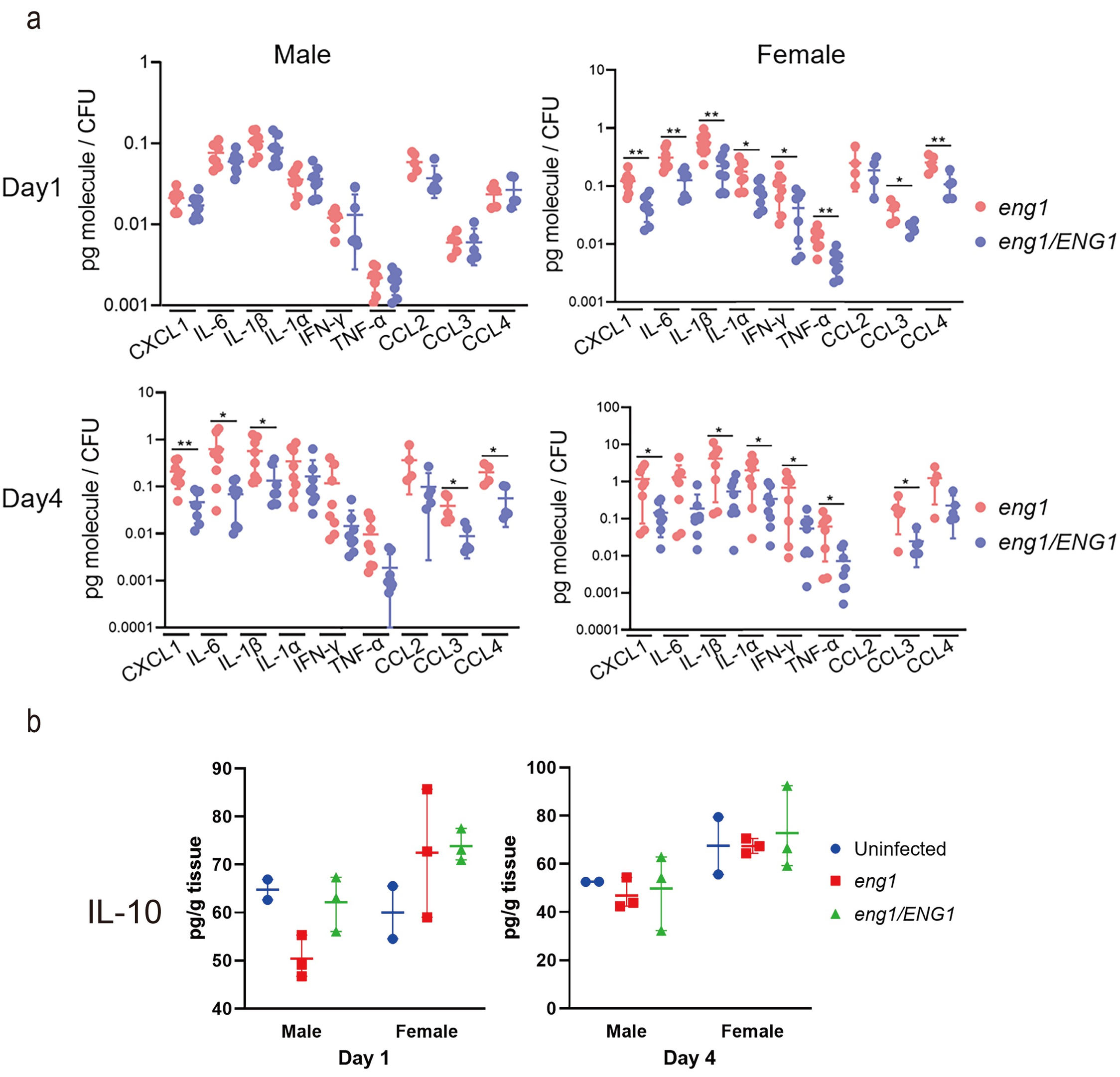

Supplement: S5 Fig — (a) Relative renal cytokine/chemokine levels at 1-day and 4-day post-infection. (b) Levels of renal IL-10 are bellow or similar to that of uninfected controls. (TIF) [file ppat.1010192.s005.tif]

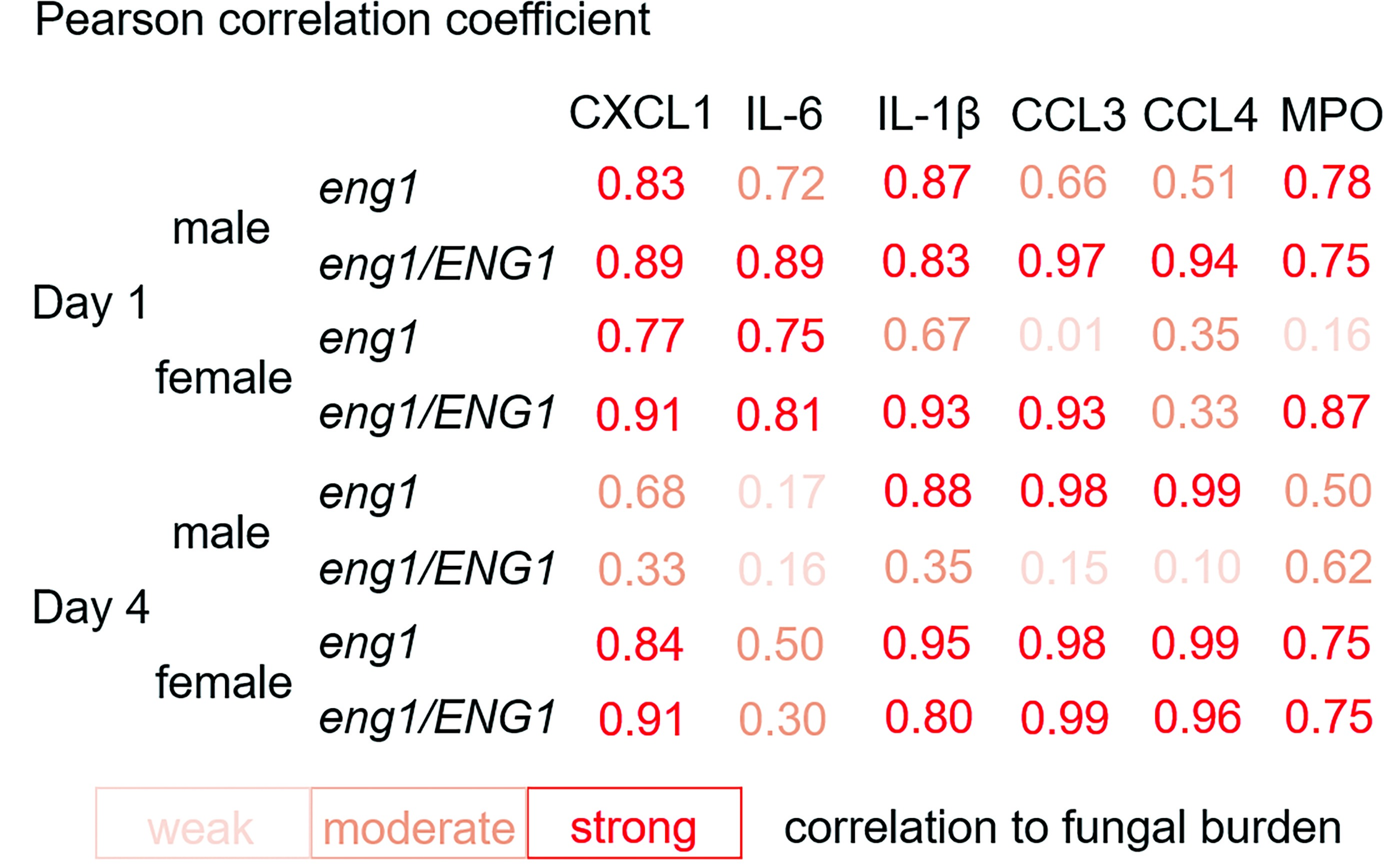

Supplement: S6 Fig — (TIF) [file ppat.1010192.s006.tif]

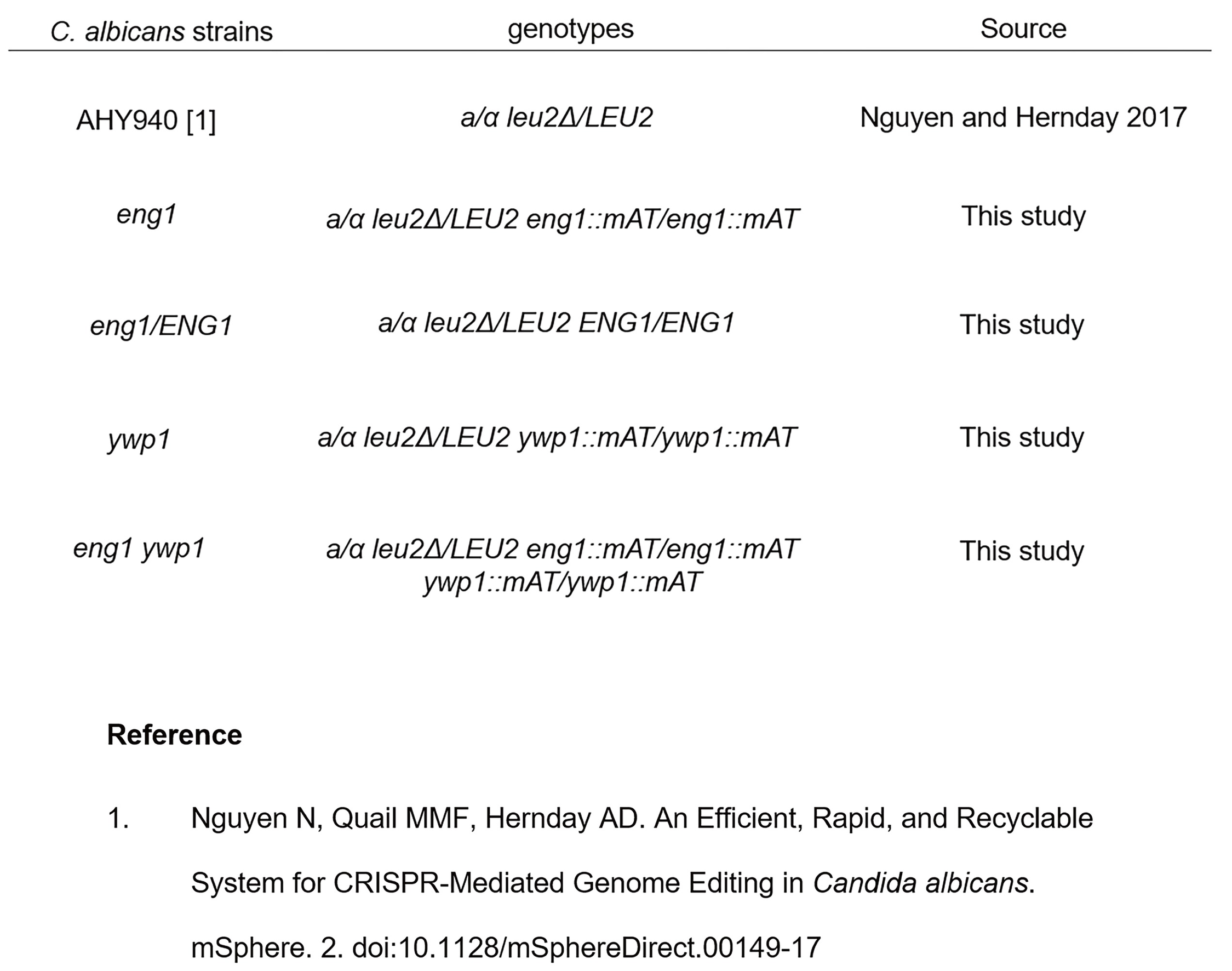

Supplement: S1 Table — (TIF) [file ppat.1010192.s007.tif]
